# Cell-Type Specific Four-Component Hydrogel

**DOI:** 10.1371/journal.pone.0086740

**Published:** 2014-01-27

**Authors:** Timo Aberle, Katrin Franke, Elke Rist, Karin Benz, Burkhard Schlosshauer

**Affiliations:** Naturwissenschaftliches und Medizinisches Institut an der Universität Tübingen, Reutlingen, Germany; Centro Cardiologico Monzino, Italy

## Abstract

In the field of regenerative medicine we aim to develop implant matrices for specific tissue needs. By combining two per se, cell-permissive gel systems with enzymatic crosslinkers (gelatin/transglutaminase and fibrinogen/thrombin) to generate a blend (technical term: *quattroGel*), an unexpected cell-selectivity evolved. QuattroGels were porous and formed cavities in the cell diameter range, possessed gelation kinetics in the minute range, viscoelastic properties and a mechanical strength appropriate for general cell adhesion, and restricted diffusion. Cell proliferation of endothelial cells, chondrocytes and fibroblasts was essentially unaffected. In contrast, on quattroGels neither endothelial cells formed vascular tubes nor did primary neurons extend neurites in significant amounts. Only chondrocytes differentiated properly as judged by collagen isoform expression. The biophysical quattroGel characteristics appeared to leave distinct cell processes such as mitosis unaffected and favored differentiation of sessile cells, but hampered differentiation of migratory cells. This cell-type selectivity is of interest e.g. during articular cartilage or invertebral disc repair, where pathological innervation and angiogenesis represent adverse events in tissue engineering.

## Introduction

Hydrogels have become attractive scaffolds in order to realize in vitro diagnostics, preformed medical products, tissue glues or cell-bearing implants to compensate organ traumata [1]. Originating from one or several components, three-dimensional matrices are formed that consist of physically associated or chemically cross-linked hydrophilic polymers with a water content of up to 99.9% [2], [3]. This significant water content provides a degree of flexibility similar to native tissues. Synthetic hydrogels, both inert and resorbable based on poly(hydroxyethylmethacrylic)acid (polyHEMA) and poly(vinyl alcohol) (PVA) partially in combination with poly(ethylene glycol) (PEG), are employed for soft contact lenses or as wound dressings. Additives improve specific performances, such as the inclusion of hydroxyapatite nanoparticles into PEG gels to address bone defects [4], [5], [6], [7]. Engineered coiled-coil or beta-sheet protein domains grafted onto synthetic polymer backbone [8] and poly-N-isopropylacrylamid (P-NIPAM)-hydrogels respond to temperature changes. Such features allow hydrogels to be used as sensors, actuators, or for controlled drug release. However, accompanying disadvantages, such as low mechanical stability, restricted gelation and slow response characteristics to external stimuli, remain to be addressed.

Biopolymers such as human albumin, deacetylated chitin, highly charged hyaluronic acid or evolutionary conserved collagen have been stabilized by chemical crosslinkers, herbal genipin, or transacting enzymes [2], [9], [10]. For medical products it is most advised to apply components with already existing approval for human application/use, such as gelatin/transglutaminase or fibrinogen/thrombin. Gelatin used here is derived from porcine skin extraction leading to partial denaturation of the native triple helix of the collagen protein. The resulting molecular structure of gelatin, including the isoelectric point around 8.5, provides colloidal solutions at physiological pH which are used to generate thermoreversible gels with a strength of 300 bloom. For long term stabilization, typically chemical crosslinkers are employed [11], [12]. To circumvent potential toxic side effects of highly reactive aldehyde-based crosslinkers, recombinant microbial transglutaminase (mTG) has been shown to be a very effective substitute. mTG has been approved for human ingestion and is widely used in nutrition processing. mTG causes protein crosslinking by transamidation between a deprotonated lysine donor residue and an acceptor glutamine [13]. The gel network is accessible to degradation by cellular metalloproteinases. Fibrinogen is a major component of the coagulation cascade involved in blood clotting. The homodimer fibrinogen is most effectively processed by the protease thrombin leading to fibrin with low solubility and a high tendency to aggregate. Under traumatic conditions, initial fibrin aggregates become crosslinked by tissue transglutaminase (factor XIIIa) to form fibrin clots which counteract bleeding. Fibrin clots can be readily solubilized by liver-derived plasmin which originates from plasminogen proteolytically activated by tissue plasminogen activator (tPA) and urokinase.

In situ crosslinked hydrogels, which provide a cell-permissive, integrative implant-tissue interface restoring tissue continuity, could represent regenerative scaffolds for regenerating neurons after prostatectomy and subsequent impotency [14], [15], and thus, provide an alternative to solid nerve guides [16], [17]. Since distinct cells beneficially remodel exogenous matrices during regeneration and inherently optimize their growth substratum, we aimed to integrate cleavage sites into scaffolds to foster axonal infiltration. Because fibrinolytic tPA/urokinase in addition to metalloproteinases is expressed in axons, we combined the gelatin- and the fibrin systems to create a quattroGel. To our surprise the resulting hydrogel abrogated axonal growth but displayed a cell-type preference potentially promising for other medical needs. Since neurite extension is based on cellular motility, we questioned how sessile and other migratory cells would respond to quattroGel. Whereas migratory cells seemed to be inhibited, non-migratory chondocytes differentiated in quattroGels.

## Materials and Methods

The following components were employed: pig skin gelatin (GELITA AG, Eberbach, Germany; Lot No. 325109), bacterial enzyme transglutaminase (N-Zyme BioTec GmbH, Darmstadt, Germany Product No. T014, Lot No. 1007T014); fetal calf serum (FCS), Hanks balanced salt solution (HBSS), phosphate buffered saline (PBS), penicillin/streptomycin (pen/strep), gentamycin and L-glutamine (all from PAA, Linz, Austria); DNA/nucleus stain 4,6-diamidino-2-phenylindol (DAPI), human type I fibrinogen from plasma (F3879), laminin (LN), L-ascorbate-2-phospate, nerve growth factor, pig skin gelatin (G2500, Batch# 128K0066), and thrombin from bovine plasma (T4648), FD&C blue #1 (861146) (all from Sigma-Aldrich, Deisenhofen, Germany); calcein (Molecular Probes Inc., USA); poly-D-lysine (PDL) and Dulbecco’s Modified Eagle Medium (DMEM) (BioWhittaker, Verviers, Belgium). Vascular endothelial growth factor (VEGF) was from PeproTech EC (recombinant human VEGF_165_, London, UK); Matrigel™ (BD Biosciences, USA).

### Methods

#### Human specimens

All clinical investigations were conducted according to the principles expressed in the Declaration of Helsinki. Cartilage tissues originated from four osteoarthritis (OA) patients aged 60, 69, 76, and 78 years (2 females, 2 males) who underwent total endoprosthetic knee replacement. Cartilage tissues were obtained from the BG Trauma Clinic in Tuebingen (Germany). The studies (# 474/2009 B02) were approved by the local ethics committee (*Ethik Kommission an der Medizinischen Fakultät der Eberhard-Karls-Universität und am Universitätsklinikum, Gartenstr. 47, D-72074 Tübingen, Germany*) and written informed consent was obtained from all individuals.

#### Animals and implantations

The maintenance and experimental manipulations of rats and mice followed the principles of laboratory animal care and the guidelines of the European Communities Council as well as German animal protection laws. Experiments were approved by the governmental review committee (*Regierungspräsidium Tübingen, Germany*; # NMI 1/10,). Rats (Lewis and Sprague Dawley, female, 12–15 weeks old, approximately 230 g) and albino mice (BalbC, female, 6–8 weeks old, approximately 30 g), both from JANVIER (S.A.S., St Berthevin Cedex, France), were held in Makrolon cages (type IV) using standard bedding (ssniff Spezialdiäten GmbH, Soest, Germany). The animal facility parameters were: 12 h/12 h light/dark intervals, 22±2°C ambient temperature, and 55±5% air humidity. Standard fodder (ssniff Spezialdiäten GmbH, Soest, Germany) and water were provided ad libitum.

Subcutaneous injections of hydrogels (see below) were performed with the aid of syringes (19 G×1″) under anesthesia (10 mg/kg Ketamin, 1 mg/kg Xylazin). Eight mice (four per gel type) received 100 µl hydrogel each on the left and right back site near the blade bones. To allow unaffected polymerization, the syringe needles were left in place for 5 min before removal. After a postoperative period of 3 months, the animals were euthanized for macro- and microscopic investigations.

#### Hydrogels

Stock solutions: a) 9% **Gelatin stock** solution was made from gelatin granules dissolved in a two-step process (30 min, 37°C and subsequently 2 h, 50°C) in cell culture medium (see below); b) **Transglutaminase stock**: the cross-linking enzyme was dissolved in PBS (7,5 U/ml); c) **Fibrinogen stock:** 10 mg/ml PBS; d) **Thrombin stock**: 200 U/ml in aqua dest and HBSS without Ca^2+/^Mg^2+^ (1∶1). The stock solutions b) and d) were sterile filtered and all stock solutions were stored for a maximum of one week at 4°C. To produce gelatin gels, gelatin and transglutaminase stocks were mixed in a 4+1 ratio. For quattroGels two-component stocks were made: e) **Enzyme stock**: 8 µl thrombin stock plus 40 µl transglutaminase stock, and f) **Matrix stock:** 300 µl gelatin stock plus 150 µl fibrinogen stock. For immediate use of quattroGels, 240 µl prewarmed (15 min, 37°C) matrix stock were added to 48 µl enzyme stock. Mixing was vigorous and rapid with the aid of a pipette but without any bubble formation. For matrix distribution analysis, Alexa488 labeled fibrinogen (1,5 mg/ml, 30% w/w with fibrinogen stock; Life Technologies, Darmstadt, Germany, F13191) and 0.3×10^9^ latex beads/ml final gelatin stock (fluorescent red, carboxylate-modified polystyrene, 2.0 µm diameter; Sigma-Aldrich, Deisenhofen, Germany, L3030) were employed. The resulting solutions were processed as above. For intense blue staining of hydrogels, FDC blue#1 (2,4 µg/ml in gelatin stock; Sigma-Aldrich, Deisenhofen, Germany, 861146) was used.

#### Osmolarity

To determine the osmolarity (mmol/kg) of all solutions necessary for hydrogel production, 10 µl of the corresponding solutions were transferred onto filter papers which were evaluated in an osmometer (VapoPressureOsmometer, Wescor, USA). Solutions were prepared three times independently and were measured twice for each experiment.

#### Diffusion analysis

For analyzing the diffusion rate through hydrogels, the passage of phenol red was monitored using a two chamber system (MINUCELLS and MINUTISSUE Vertriebs GmbH, Bad Abbach, Germany) with phenol red containing DMEM cell culture medium in the upper chamber and PBS in the lower chamber as described earlier [12]. As interface between both chambers, control membranes or hydrogels were clamped. Positive control: gauze (20 µm pores); negative control: parafilm. Hydrogels were placed on gauze to facilitate handling: 150 µl gel solution on Ø 1.4 cm gauze. Diffusion intervals tested were: 2, 4, 6, 8, 16, and 24 h at 37°C. The absorption of phenol red was measured spectrophotometrically at 558 nm (Lambda Bio+, Perkin Elmer, Waltham, USA). The permeability index was calculated according to the formula *Pe = dx/dt*×*(C*×*A)−1* (Pe - permeability coefficient (cm/s), dx/dt - the rate of translocation (pmol/s), C - initial dye concentration in the donor chamber (pmol/cm^3^), A - area of penetration (cm^2^). For the *Pe* calculation, absorption values measured after 2 h were used.

#### Rheological measurements

A Kinexus rheometer (Malvern, USA) with 20-mm parallel plates at a distance of 0.6 mm was used for rheometric analysis of hydrogels. Hydrogels were produced directly between the plates to start rheometric measurements instantaneously. For amplitude sweep tests, the rheometer was maintained at 37°C. The storage modulus G′ was recorded at 1 Hz with a constant normal compressive force of ∼ 5 g (0.05 N). The amplitude of the deformation (y) was continuously increased (deformation: 0.1–100%). Gelation measurements were carried out with an amplitude of deformation of 1% and at 37°C for 2 h at 1 Hz. For frequency sweep analysis, G′/G″ were recorded at increasing frequencies (0.1 Hz –10 Hz) with a constant amplitude of deformation of 1%.

#### Cell cultures

L929 cells (immortalized fibroblasts from mouse connective tissue; DSMZ: Braunschweig Germany; German Collection of Microorganisms and Cell Cultures GmbH) were cultured in RPMI (Roswell Park Memorial Institute) 1640, 10% heat inactivated FCS (fetal calf serum), 2 mM L-glutamine, 100 U/ml penicillin, 10 mg/ml streptomycin. Human umbilical vascular endothelial cells (HUVEC, passage 3–7) (PromoCell, Heidelberg, Germany) were cultured (95% air/5% CO_2_; 37°C) in growth medium (ECGM with supplement mix (PromoCell, Heidelberg, Germany) and 50 U/ml penicillin-G/50 µg streptomycin). For cell passage, DetachKit 30 (PromoCell, Heidelberg, Germany) was used. Primary human articular chondrocytes (passage 1 and 2), isolated as described previously [10], were cultured in DMEM/Ham’s F-12 (2∶1; Biochrome, Berlin, Germany); 10% FCS, 100 U/ml penicillin-G, 100 µg/ml streptomycin; 150 nmol/ml L-ascorbate-2-phosphate (Sigma Aldrich Chemie GmbH, Deisenhofen, Germany, A8960), humidified atmosphere, 95% air, 5% CO_2_. For culturing dorsal root ganglia (DRG, dissected from rat embryos E17/18) the following medium was employed: DMEM-B27-Medium, 10% MEM Earles, 1% pyruvate, 1% L-glutamine (2 mM), 4% NaHCO_3_, 3% glucose, 2% B27 supplement, and 25 ng/ml nerve growth factor (NGF, Sigma-Aldrich Chemie GmbH, Steinheim, Germany). Explanted DRGs were cultured on PDL (50 µg/ml, 1 h, 37°C)/laminin (20 µg/ml, 1 h, 37°C) coated coverslips, or on top of, or incorporated into hydrogels for up to 3 days. Media were purchased from PAA Laboratories GmbH, Pasching, Austria, Life Technologies, Carlsbad, USA, and Invitrogen, Darmstadt, Germany.

#### BrdU-labeling

For metabolic BrdU-labeling of proliferating cells, the 5-Bromo-2′-dU Labeling/Detection Kit I (Roche Diagnostics GmbH, Mannheim, Germany) was employed. Cells were cultured on top of the hydrogels for 48 h, or as control on uncoated (L929) or glutaraldehyde crosslinked gelatin coated coverslips (HUVEC, chondrocytes). The incubation period with BrdU-Labeling Reagent was 5 h (L929) or 24 h (HUVEC, chondrocytes).

#### Tube formation

In order to monitor tube formation, HUVEC were cultured on hydrogels with media supplemented with 1.5 ng/ml VEGF. As positive control, HUVECs, and, as negative control, human foreskin fibroblasts (gift of Prof. P. Rodemann, University Tübingen, Germany) were cultured on matrigel™ (Becton Dickinson GmbH, Heidelberg, Germany).

#### Vitality of cells in gels

Vitality quantification of incorporated cells after three days was performed by a life/dead-staining with calcein and DAPI. Image stacks were collected using Apotome^©^-microscopy (Carl Zeiss AG, Oberkochen, Germany) and data analysis was performed with *ImageJ.*

#### Gene expression analysis

For total RNA extraction, hydrogels with incorporated cells were solubilized (collagenase B, Roche Diagnostics GmbH, Mannheim, Germany, 11088807001, 4 mg/ml, 45 min., 37°C), centrifuged, and the cell pellet lysed in RLT-buffer (Quiagen, Hilden, Germany). RNA was extracted using the RNeasy mini kit plus DNase I digestion (Qiagen). RNA purity was determined by photometric measurement of the 260 nm/280 nm ratio (Bio Photometer, Eppendorf #613125116, Hamburg, Germany). Complementary DNA (cDNA) was synthesized (Reverse Transcriptase Core kit (Eurogentec, Cologne, Germany) with EuroScript reverse transcriptase (Moloney Murine Leukemia Virus reverse transcriptase, 50 U/µl) and oligo-dT primers. Reverse transcription was performed in a total volume of 50 µl at 48°C for 30 min in a thermocycler (Whatman Biometra, Gottingen, Germany). Gene expression was analyzed by quantitative real-time PCR (Applied Biosystems 7500 Fast Real-Time PCR System) employing qPCR mastermix plus SYBR green I (low ROX) kit (Eurogentec). To 20 µl of total cDNA (approximately 20 ng –100 ng), 100 - 200 mM primers (according to the optimal condition for each primer) and the 2×reaction buffer were added to a total volume of 50 µl. Primers were designed with the primer express 2.0 software (Applied Biosciences, Darmstadt, Germany) and purchased from BIOTEZ (Berlin, Germany). Glyceraldehyde-3-phosphate dehydrogenase (GAPDH) was used as the internal standard. Primer sequences: GAPDH AGAAAAACCTGCCAAATATGATGAC (reverse) and TGGGTGTCGCTGTTGAAGTC (forward), collagen type I GCTGGCAGCCAGTTTGAATATAAT (reverse) and CAGGCGCATGAAGGCAAGT (forward), collagen type II AGAGGTATAATGATAAGGATGTGTGGAAG (reverse) and GTCGTCGCAGAGGACAGTCC (forward), PCR reaction parameters were: 95°C, 10 min, 40 cycles of 95°C for 15 sec, 60°C for 30 sec, and 72°C for 30 sec. A melting curve was generated after the last cycle. Threshold cycles (Ct values) were determined for each gene using Sequence Detection System software (Applied Biosystems). PCR efficiencies were calculated for each primer pair using a calibration curve. Relative gene expression was calculated according to Benz et al. [10].

#### Histological stainings

Mouse tissues and implants were fixed (4% PFA/PBS, 22°C, 2 h) and immersed in increasing concentrations of sucrose/PBS (10%, 20%, 30%, 22°C, 2 h), whereas, for structural analysis, gels without cells were quick-frozen in liquid nitrogen without fixation and sucrose immersion. Both specimen types were cryosectioned (10–30 µm slices; Cryostat Leica CM3050S, Leica Microsystems, Wetzlar, Germany), washed (H_2_O, 1×1 min), stained with hematoxylin (8 min), rinsed (running tap water, 10 min), and washed (H_2_O, 1×5 min). Thereafter, specimens were counterstained with 0.2% eosin (4 min), washed, dehydrated (increasing alcohol series (70% ethanol, 1 min, 96% ethanol, 1 min, 100% ethanol, 2×1 min, 100% ethanol:roti-histol (1∶1), 2 min, roti-histol, 2×2 min), mounted in Neo-Mount Medium (Merck, Darmstadt, Germany), and stored at 4°C.

#### Immunocytochemistry

For fluorescence staining, implants, DRG explants, cells grown on coverslips or hydrogels, or cells embedded in hydrogels were fixed (4% PFA/10% sucrose/PBS, 22°C, 30 min), washed (PBS, 3×5 min), permeabilized (0.02–0.1% Triton X-100/PBS; 15 min), and blocked (1% BSA/5% normal goat serum/PBS, 1 h). Primary antibodies (SMI31 against neurofilament (Sternberger Monoclonals, USA; 1∶1000 in 1% BSA/PBS) were applied for 1–2 h/22°C, or 16 h/4°C. After washing 3x with PBS, the secondary antibody was added (Cy3-labeled goat anti mouse: 1∶250 in 1% BSA/PBS, 1–2 h, 22°C, in the dark; Dianova, Hamburg, Germany) and washed. Cell nuclei staining was performed with 5 µg/ml DAPI (4′,6-diamidine-2-phenylindol) in PBS, (10 min, 22°C, in the dark). Alternatively, specimens were processed for actin staining (phalloidin oregon green or red (1∶50/PBS, 3 h, 22°C; Invitrogen, Darmstadt, Germany), washed, covered with fluorescence mounting medium, and microscopically analyzed (Axiovert 200 M and CLSM, Carl Zeiss AG, Oberkochen, Germany). Vital staining of cells in vitro was achieved by incubation with calcein and DAPI (0.1 µg/ml Calcein, 2.5 µg/ml DAPI, 10 min, 37°C). Image analysis was performed with *ImageJ*.

#### Statistics

Statistical analysis was based on at least three independent series of experiments with doublets per series, using one-way ANOVA followed by a Tukey HSD posttest comparison. Data not following Gaussian distribution were analyzed by Kruskal–Wallis one-way analysis of variance. Data (mean ± s.d.) were considered to be statistically significant when the p-value was p<0.05.

## Results

A cell-permissive gelatin gel and a gelatin gel plus fibrin at the physiological concentration as evident in the blood (termed quattroGel) were synthesized and compared.

### Microstructure of Hydrogels

**Figure 1:**
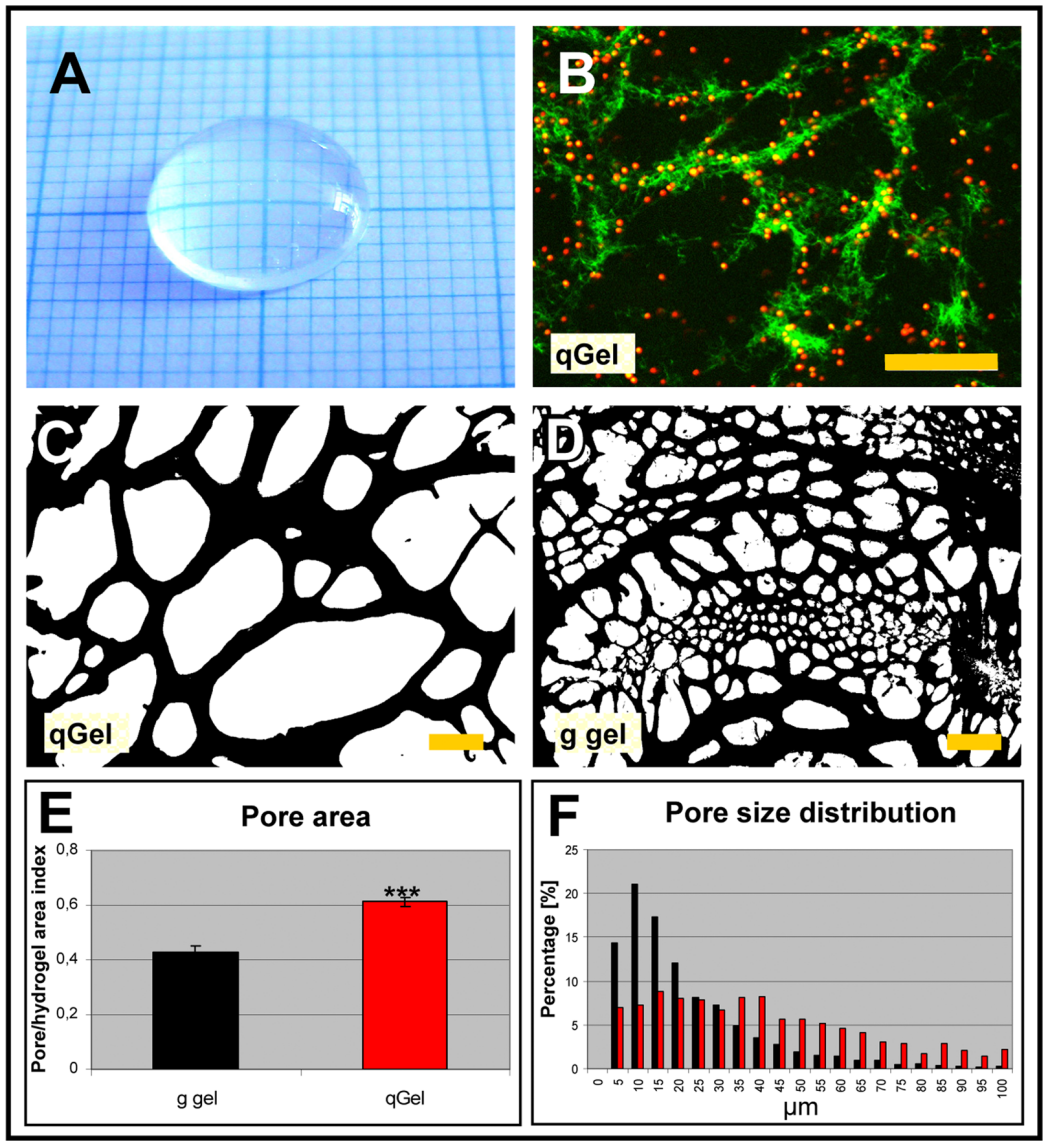
Microstructure of hydrogels. (A) The quattroGel forms a macroscopically homogeneous hydrogel. (B) quattroGel with incorporated Alexa488-fibrin and fluorescent latex beads in the gelatin solution. Fibrin and gelatin appear to be equally distributed within quattroGel lamellae. Green – fluorescently labeled fibrin; red – labeled latex beads in gelatin. (C, D) HE stained cryosections of quattroGel (C) and gelatin gel (D). (E, F) Quantification of pore area (E) and pore distribution (F) in both gels indicated a higher porosity in quattroGel. qGel – quattroGel; g gel – gelatin gel. Scale bars: (B) 50 µm, (C and D) 20 µm.

Since the microstructure could influence the diffusion characteristics of nutrients and cell interactions, we analyzed gel architectures and monitored potential temporal changes during incubation. Cryosections of quick-frozen hydrogels were investigated directly 1 h after polymerization or after 2 and 7 days while being exposed to in vitro culture medium. Hematoxylin/eosin stained sections visualized the porous character of hydrogels (Fig. 1C, D), though macroscopically both gels appeared homogeneous and translucent (Fig. 1A). Both gels displayed sponge-like morphology characterized by a random distribution of major and minor cavities and lamella branches which were stable under physiological conditions (37°C, PBS pH 7.2) over the test period of one week. Quantification of the pore areas revealed higher values for quattroGels (61%) in comparison to gelatin gels (43%) (Fig. 1E). In parallel, the average pore size of 58 µm for quattroGels was significantly higher in contrast to gelatin gels with 27 µm. As depicted in Fig. 1F, the pore size distribution was shifted to bigger size populations in quattroGels. To analyze the molecular distribution of both matrix components, fluorescently labeled fibrin and gelatin solution with counter-coloured latex beads were applied. Both labels appeared equally distributed in the gel lamellae suggesting that fibrin and gelatin mix without the formation of microdomains in the scaffold (Fig. 1B).

### Viscoelastic Properties

**Figure 2:**
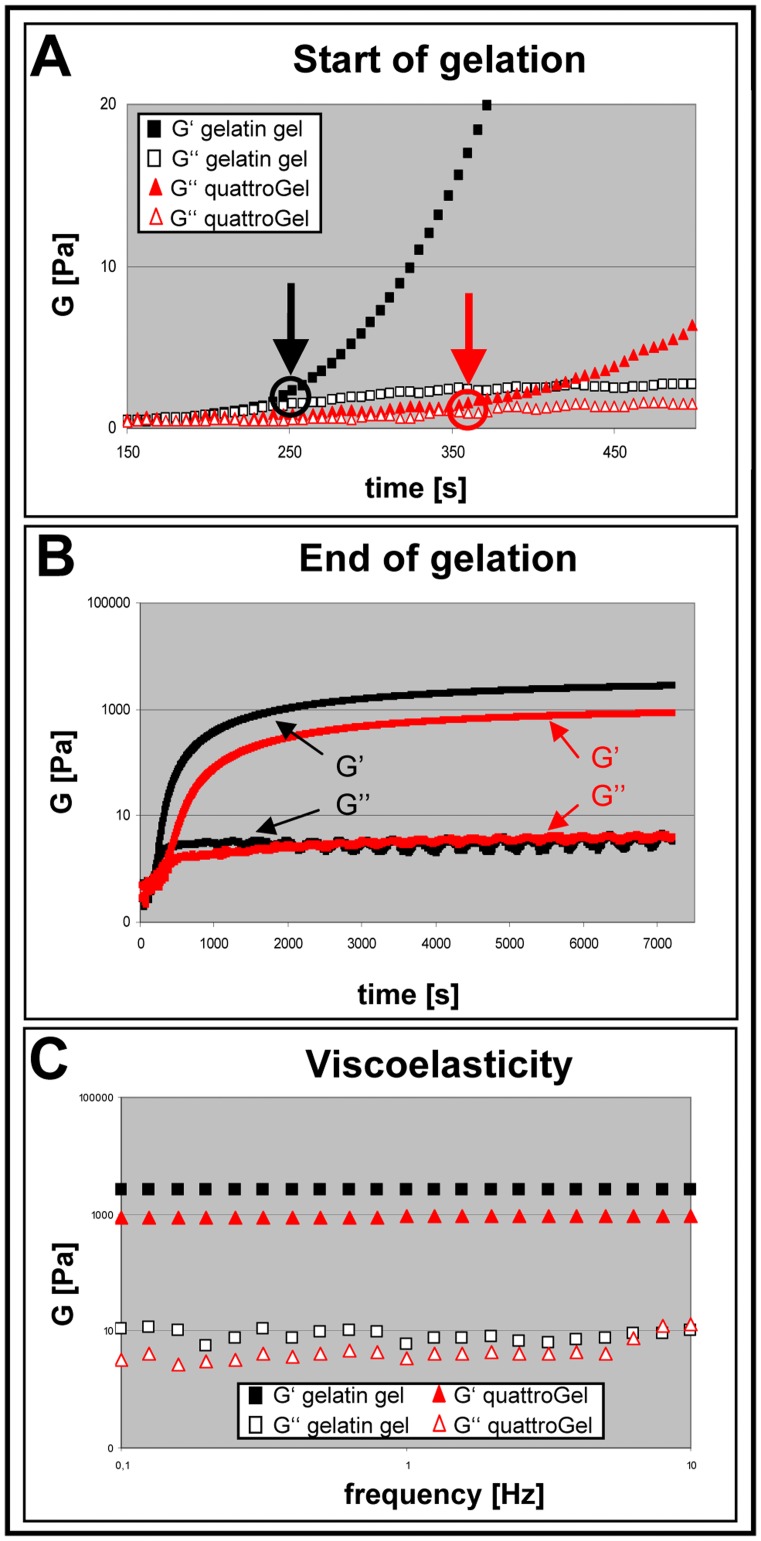
Viscoelastic properties. (A) Start of gelation. Gelation points deduced from crosspoints of G′ and G″ (circles with arrows); quattroGel showed a delayed gelation (360 sec) compared to gelatin gel (252 sec). (B) End of Gelation. *Oscillatory time sweeps* to unravel gelation kinetics of the gelatin gel (black) and quattroGel (red) over 2 h at 37°C. Both gels showed typical asymptotic graphs with gelation essentially finalized after about 1 h (plateau phase). (C) Viscoelasticity. *Frequency sweep* after curing of the gels revealed semi-rigid, elastic properties for both gels as deduced from storage moduli (upper curves) two orders higher than the corresponding loss moduli (lower curves). n = 3.

To evaluate the time sequence of gelation and the resulting elasticity, rheometric measurements were performed by recording the parameters G′ (storage/elastic modulus) and G″ (loss/viscous modulus). G′ represents the elastic storage of energy, and is a measure of how well-structured a hydrogel is. G″ represents the viscous energy dissipation and changes depending on the viscosity of the hydrogel [18]. An amplitude sweep test was performed to determine the linear viscoelasticity (LVE) range. For subsequent experiments a 1% deformation was chosen (the LVE range ended between 80–100% deformation (data not shown). Gelation (circles/arrows in Fig. 2A: crosspoints of G′- and G″ curves) of the gelatin gel started after 252 seconds, and of the quattroGel after 360 seconds (37°C) (Fig. 2A). As revealed by an *oscillatory time sweep* test both hydrogels displayed a proper gelation approaching a stable plateau phase after about an h (Fig. 2B). The gelation at 37°C resulted in final storage modulus values of 2.7 kPa (gelatin gel) and of 0.85 kPa (quattroGel). Next, a frequency sweep test was performed providing a ‘fingerprint’ of the viscoelastic hydrogels under non-destructive conditions [19], [20]. Since both G′ values were two orders higher than the corresponding G″, both hydrogels are more elastic than viscous. G′ values were constant under increasing frequency indicating that both hydrogels had the ability to resist structural changes under strain. In conclusion, both hydrogels displayed proper gelation and viscoelastic properties potentially applicable in regenerative medicine.

### Diffusion Properties

**Figure 3:**
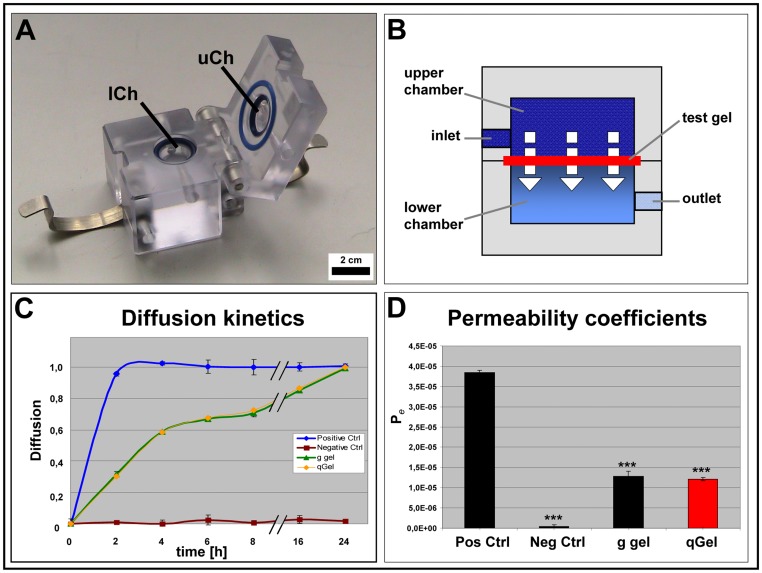
Diffusion characteristics. (A, B) Two-chamber device and scheme. Test gels were inserted as interface between the upper chamber (uCh) containing a marker chromophore and the lower chamber (lCh) filled with colourless buffer. (C) Diffusion kinetics over 24 h with equilibrium reached after one day for both hydrogels. Negative control (Neg Ctrl): parafilm; positive control (Pos. Ctrl: gaze. (D) Permeability coefficients. Gelatin gel (g gel) and quattroGel (qGel) displayed a similar, restricted diffusion when compared with the positive control. n = 3, s.d., p<0.001 compared with positive control.

To assess the permeability of hydrogels for potential cell incorporation, the diffusion of phenol red was analyzed in a two chamber system. The upper chamber was filled with phenol red containing medium and the lower chamber was filled with PBS. The opening separating the two chambers harbored either gauze (20 µm pores) as positive control, parafilm as negative control, or hydrogels placed on gauze to facilitate handling (Fig. 3A, B). In positive controls rapid stain diffusion within 2 h was evident, resulting in nearly equal absorption values in both chamber compartments. In contrast, no diffusion was detectable in negative controls during the 24 h test period. Both hydrogels displayed similar diffusion curves. Equilibrium was not achieved before 24 h (Fig. 3C). For defined comparison, the permeability coefficients (P*_e_*) were calculated. Whereas the positive control exhibited a P*_e_* of 3.84×10^−5^ cm/s, both hydrogels had coefficients of 1.2×10^−5^ cm/s (Fig. 3D).

The calculated osmolarities of both hydrogels were in the physiological range (gelatin gel: 315.23 mmol/kg, quattroGel: 321.49 mmol/kg) and therefore appropriate for subsequent cell cultures.

### Biocompatibility in vitro

**Figure 4:**
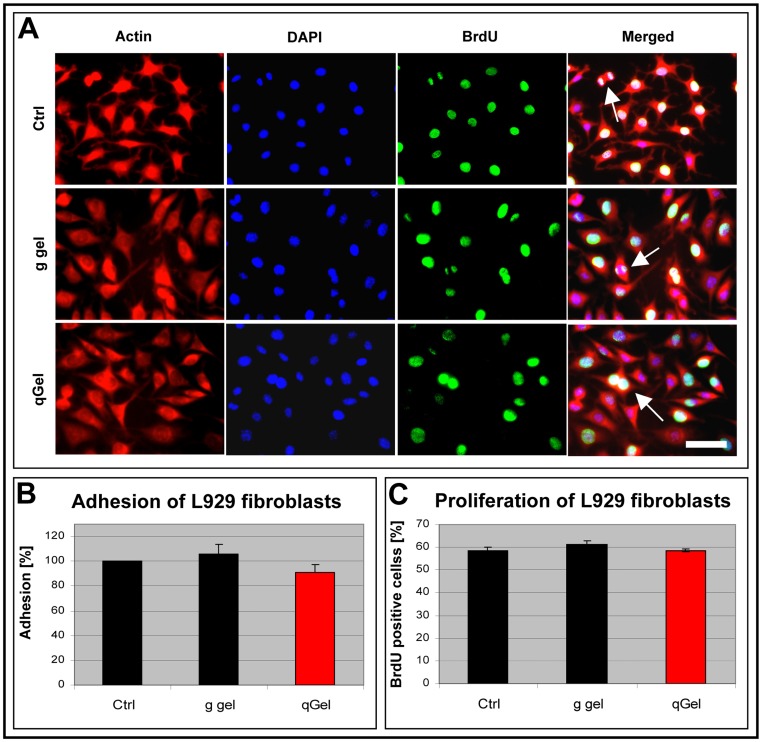
Biocompatibility in (A) The cell line L929 was cultured on a positive control substrate (Ctrl), on gelatin gel (g gel) and on quattroGel (qGel), metabolically marked with bromodeoxyuridine (BrdU, green) to identify mitotic cells and cytochemically labeled with fluorescent phalloidin (actin, red) and DAPI (blue) to display all cell nuclei. Pictures of individual rows represent corresponding images. Arrows mark cells in different stages of mitosis. Quantification of cell adhesion (B) and cell proliferation (C) indicate that no statistically significant differences exist between different substrates. n = 3, s.d., p<0.05. Scale bar: (A) 50 µm.

To investigate principle adhesion and proliferation of cells grown on hydrogel surfaces, the mouse fibroblast cell line L929, recommended by regulatory boards e.g. for the ISO 10933 for cytotoxicity assays, was chosen. Cells were plated onto hydrogels or coverslips as positive control and cultivated for two days, exposed to metabolic DNA labeling, and double stained for BrdU and actin-binding phalloidin to visualize both cell morphology and proliferation. Fibroblasts seemed to develop normal phenotypes when compared to cells grown on the control substrate (Fig. 4A). Quantification of cytochemically stained micrographs showed no significant difference of cell adhesion between hydrogels and the positive control substrate (Fig. 4B). Similarly, fibroblast proliferation on both hydrogels was in the same range as in positive controls (Fig. 4C). In summary, both hydrogels were biocompatible and represented permissive cell substrates without significant differences.

### Neuronal Outgrowth on Hydrogels

**Figure 5:**
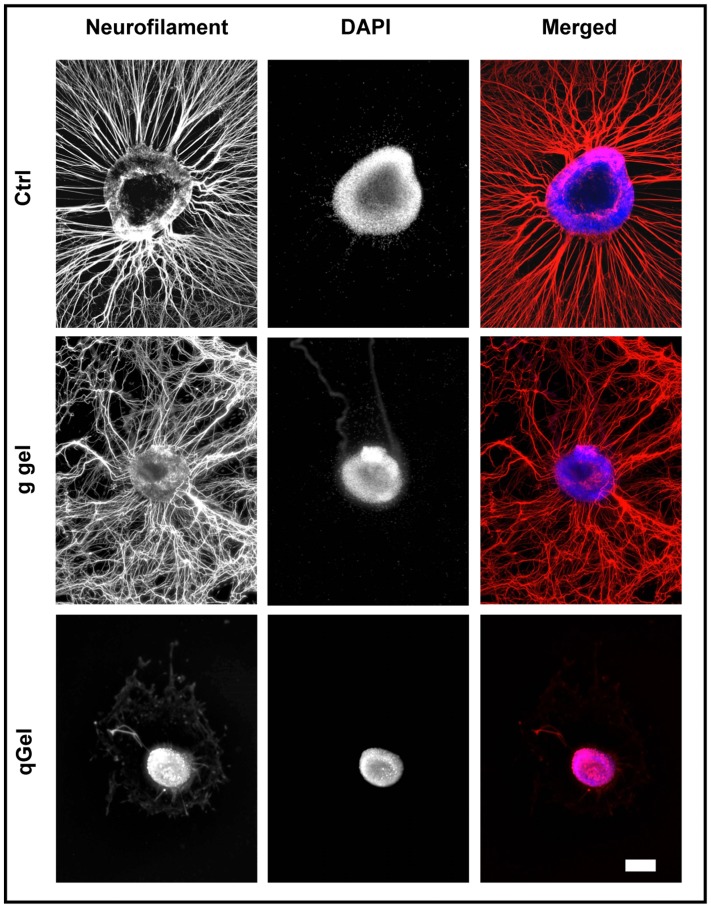
Neurite growth. Rat dorsal root ganglia were explanted on a positive control substrate (Ctrl, PDL/laminin), on gelatin gel (g gel), and on quattroGel (qGel) and after culturing immunostained for neurites (red, neurofilament) and for cell nuclei (blue, DAPI). Significant neurite outgrowth is evident in the control and on gelatin gel, not on quattroGel. Pictures of individual rows represent corresponding images. Scale bar: 200 µm.

Due to the initial intention to synthesize a neuritotrophic hydrogel that fosters neuronal regeneration in vivo, primary cultures of the rodent peripheral nervous system were employed. Rat dorsal root ganglia (DRG) were explanted onto hydrogels and PDL/laminin-coated coverslips as positive control substrate. After cultivation, cytoskeletal neurofilaments of axons were visualized by immunocytochemistry, revealing a vigorous axonal outgrowth from DRGs grown on the control substrate (Fig. 5, first row). On gelatin gels axonal outgrowth was also evident, though less oriented and to an overall lesser extent (Fig. 5, middle row). In remarkable contrast to gelatin gels, on quattroGels DRG explants showed essentially no axonal extension, though the tissue adhered properly to the gel (Fig 5 bottom row). Therefore, despite comparable tissue adhesive properties of both gels, a negative cell specificity was suggested as deduced from neuronal inhibition on quattroGels.

### Vascular Differentiation on Hydrogels

**Figure 6:**
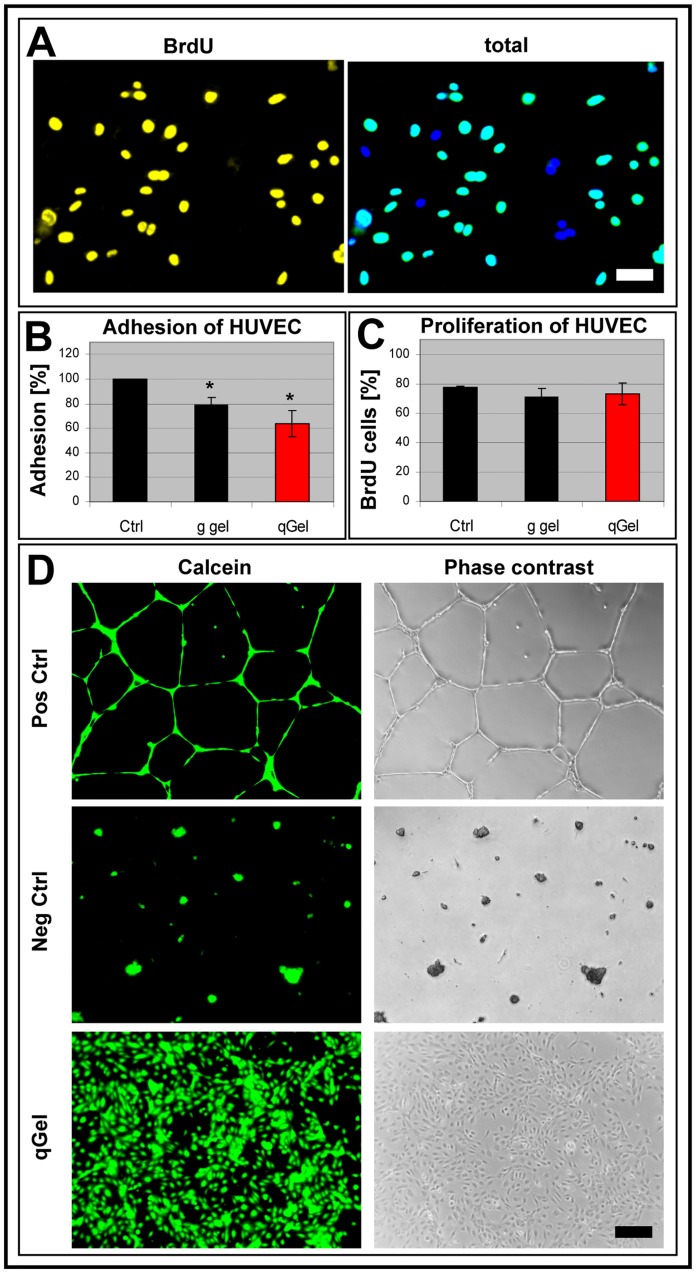
Endothelial differentiation. (A-C) Human umbilical vascular endothelial cells (HUVEC) were cultured on a positive control substrate (Ctrl), on gelatin gel (g gel) and on quattroGel (qGel), metabolically marked with bromodeoxyuridine (BrdU) to identify mitotic cells and DAPI to display all cell nuclei. (A) Corresponding false color images of HUVEC on quattroGel (left: proliferating cells; right: total cells). Quantification of cell adhesion (B) and cell proliferation (C) indicate principle cell adhesion on all substrates, though reduced on both hydrogels. No statistically significant differences exist between different substrates with regard to cell proliferation. (D) Calcein staining of cultured cells. HUVEC cultured on a positive control substrate (Pos Ctrl, Matrigel™) differentiate vessel-like tubes. No tubes are formed by fibroblasts on Matrigel™ (Neg Ctrl). HUVEC do not differentiate tubes on quattroGel. n = 3, s.d., p<0.05. Scale bar: (A) 50 µm, (D) 200 µm.

The lack of axonal outgrowth on quattroGels suggested that possibly highly motile cells and cell processes might be hindered in general. Consequently, angiogenesis moved into focus, because endothelial cells need to migrate extensively in order to form tubular structures. In a first set of experiments, human umbilical endothelial cells (HUVECs) were seeded onto hydrogels and a positive control substrate in the presence of DNA labeling nucleotide BrdU incorporated by mitotic cells. Subsequent immuno double staining allowed us to distinguish between the total cell population of adhering cells (blue) and proliferating cells (yellow) (Fig. 6A). Quantitative evaluation indicated that endothelial cell adhesion was evident on all three substrata, but somewhat reduced on hydrogels. The difference between both gels was not statistically significant (Fig. 6B). Proliferation of those cells that adhered was not statistically significantly different, since similar mitotic cell populations (around 70%) were recorded on all three substrates (Fig. 6C). To analyze angiogenesis in vitro, the tube formation assay was used, employing matrigel™ as positive control. Human foreskin fibroblasts, which normally do not form tubes, were employed as negative control. After vital cell staining with calcein, it became evident that on gelatin gels and quattroGels HUVECs failed to develop tube–like structures, but rather formed cell monolayers (Fig. 6D). In the assay quality controls, on matrigel™ HUVECs cultivation (positive control) led to prominent tube formation, whereas human fibroblasts (negative control) failed to do so. Thus, on quattroGel both cell types (neurons and endothelial cells) were precluded from those differentiation processes that depended on migratory activity.

### Chondrocyte Differentiation in Hydrogels

**Figure 7:**
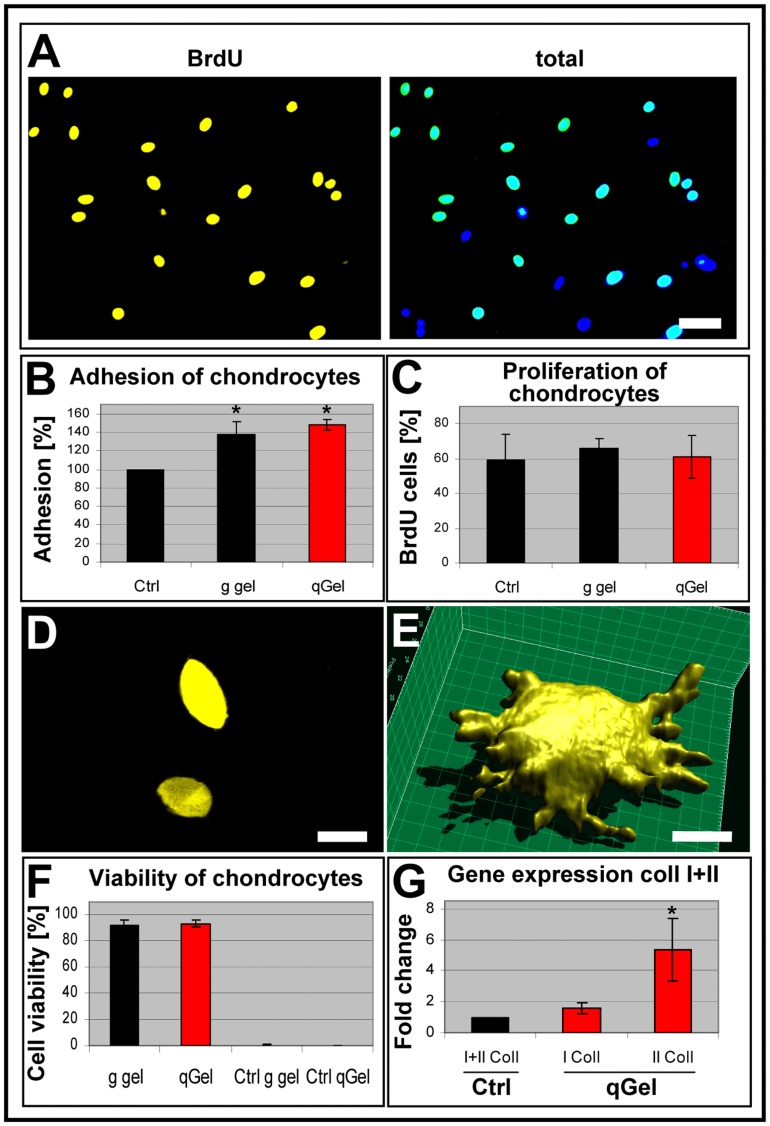
Chondrocyte differentiation. (A-C) Human chondrocytes were cultured on a positive control substrate (Ctrl), on gelatin gel (g gel), and on quattroGel (qGel), and metabolically marked with bromodeoxyuridine (BrdU) to identify mitotic cells and DAPI to display all cell nuclei. (A) Corresponding false color images of chondrocytes on quattroGel (left: proliferating cells; right: total cells). Quantification of cell adhesion (B) and cell proliferation (C) indicated principle cell adhesion on all substrates with an increased adhesion on both hydrogels. No statistically significant differences existed between different substrates with regard to cell proliferation. (D) In-depth confocal images of actin stained chondrocytes with typical ovoid morphologies within a quattroGel matrix. (E) 3-D reconstruction of a rare chondrocyte extending short processes. (F) The viability of chondrocytes incorporated into the hydrogels after 3 days was very high. For negative controls (Ctrl), specimens were exposed to detergent. (G) Gene expression analysis by RT-PCR of chondrocytes enzymatically released from hydrogels after two weeks. A chondrocyte-specific increase of type II collagen synthesis but not of type I collagen used as reference was evident. Values were compared with monolayer control cultures. n = 3, s.d., p<0.05. Scale bars: (A) 50 µm, (D) 10 µm, (E) 6 µm.

After elucidation of the migratory restriction on quattroGel, we questioned how sessile cells would interact with the hydrogels. Chondrocytes isolated from human patients were seeded onto a positive control substrate and both hydrogel types, metabolically BrdU-labeled, and quantified microscopically. Qualitative evaluation revealed that chondrocytes significantly proliferated on quattroGel, as deduced from BrdU incorporation (Fig. 7A). Quantification of cell adhesion indicated an increase of about 40% on both hydrogels compared to the positive control (Fig. 7B). The percentage of mitotic chondrocytes was similar on all three substrates (Fig. 7C). To monitor functional parameters, chondrocytes were integrated at low density into matrices in order imitate aspects of native cartilage tissue. As analyzed by confocal laser scan microscopy, cells displayed mostly ovoid to speroid morphologies with cell diameters in the range of 12–18 µm (Fig. 7D), similar to intact tissue. Only a minority of chondrocytes showed small cell protrusions (Fig. 7E). Counterstaining suggested preferential disintegration of quattroGel around these cells (data not shown). Since differential collagen isotype expression provides a meaningful measure to qualify the differentiation- versus dedifferentiation state of chondrocytes, cells that had been cultured for two weeks in quattroGel were enzymatically released from gels and processed for quantitive real-time RT PCR. Cell viability was, with about 90%, very high (Fig. 7F). Negative controls based on application of membrane destructing detergent classified the assay as being valid. Comparison of two-dimensionally cultured control cultures with those in 3D clearly indicated that type I collagen expression did not significantly change, in contrast to type II collagen which rose about 5-fold. This observation is of special interest, because type II collagen increase with concomitant constant type I collagen synthesis represented an indication of ongoing chondrocyte differentiation. The observed collagen isoform profile is in agreement with the lack of elongated fibroblast-like morphologies which would be indicative of dedifferentiated chondrocytes. In summary, the data suggest that quattroGel provided an inductive microenvironment for sessile chondrocytes.

### Longterm Performance in vivo

**Figure 8:**
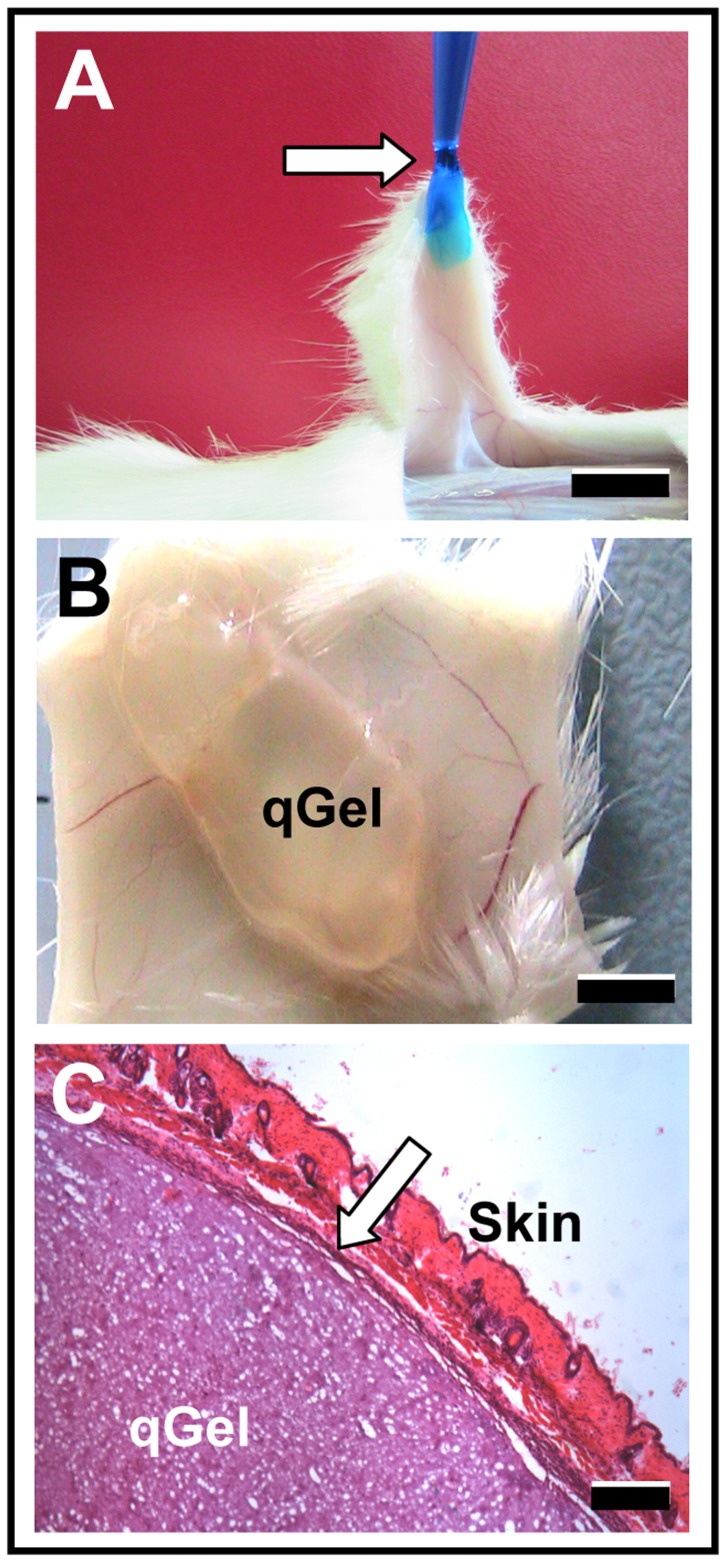
In vivo stability. (A) QuattroGel was blue-stained to improve visualization and applied to a rat skin flap. Strong adhesion (arrow) between the pipette tip with quattroGel and mouse tissue allowed the lifting of a skin flap. (B) Subcutaneous situs three months after local injection of quattroGel, which appeared to be stable without adverse clinical symptoms. (C) HE stained tissue section of a three-month implant depicting the skin, the porous quattroGel (qGel) and a thin, dark stained encapsulation layer (arrow). Scale bars: (A) 10 mm, (B) 5 mm, (C) 200 µm.

First pilot experiments in vivo aimed to reveal the principal applicability and stability of quattroGels. In situ experiments on exposed mouse skin tissue provided evidence that the hydrogel, which was labeled with FDC blue for better visualization, attached firmly to native tissue surfaces - most likely due to transglutaminase activity (Fig. 8A). In a subsequent approach, 100 µl gel solution was subcutaneously injected under the back skin of mice. Macroscopic inspection of explanted specimens demonstrated that the gels were not disintegrated within the recording period of 3 months and did not display signs of significant vascularization, inflammation, or macroscopic encapsulation at this time point (Fig. 8B) though signs of transient inflammation were evident during the initial postoperative period. Histological analysis of HE-stained tissue sections of 3-months-implants revealed the sponge-like architecture of hydrogels, and suggested a minor implant capsule of less than 50 µm (Fig. 8C). Therefore, quattroGel remained intact without significant signs of deterioration. In contrast, gelatin gels had been resorbed in the majority of cases (5 out of 8 implantation sites). Most interestingly, the softer quattroGel displayed a more extended in vivo-stability than the gelatin gel. In none of the cases was angiogenesis evident in quattroGels explanted after 3 months, which is consistent with the *in vitro* data.

## Discussion

Despite the widespread application of fibrin- and gelatin matrices together with the crosslinking enzymes thrombin and transglutaminase [9], [21], [22], [23], [24], [25], [26], [27], no reports can be found describing the combination of these components to create a four-component blend with unique biological features. Based on a conceptual error, that the integration of fibrin-based proteolytic sites into a gelatin matrix would further accelerate neuronal infiltration, we observed just the opposite and identified a cell-type specificity that could be of interest for engineering indications that aim at fostering sessile rather than motile cells.

### Hydrogel Impact on Cell Adhesion and Proliferation

Both of our hydrogels allowed - though to different degrees - principal adhesion of all six investigated cell types (fibroblasts, neurons, endothelial cells and chondrocytes - this paper, as well as epithelial and glial cells [14]). This is in agreement with a number of reports focusing on the cellular permissiveness of gelatin and fibrin matrices [28], [29], [30], [31]. That cell-to-collagen adhesion and cell functioning are not necessarily interdependent has been shown for integrin α2ß1–null fibroblasts. These cells adhere to collagen even in the absence of collagen-specific receptors, but fail to generate mechanical forces [32]. Such an adhesion-function segregation is also evident in our endothelial and neuronal cultures in which, despite cell adhesion on quattroGels, angiogenesis and neurite formation were essentially abrogated. Notably, concerning cell proliferation, both gel types were essentially indistinguishable from each other, which is consistent with published data on mitotic indices on pure gelatin and fibrin hydrogels [26], [28]. Thus, a defined combination of two permissive gels resulted in a cell-type specificity with regard to cell differentiation but leaving proliferation essentially unaffected.

### Cell Type Specificity

Abrogation of neurite outgrowth and of endothelial tube formation on quattroGels would not have been expected in the first place since both matrix components are reported to be neuritotrophic and angiogenic [24], [29], [33], [34]. In addition, fibrin in conjunction with angiogenic growth factors VEGF/FGF supports angiogenesis in wound healing paradigms in vitro and in vivo, whereas the impact of type I collagen (the native precursor protein of major gelatin components) has remained controversial. Rao et al. (2012) find increased vascular network formation in collagen/fibrin matrices supplemented with mesenchymal stem cells [35], and human dermal microvascular endothelial cells are reported to form angiogenic sprouts on fibrin but not on mixed collagen-fibrin hydrogels (1∶1 ratio) [36]. The latter is in agreement with our results based on human umbilical endothelial cells on enzymatically crosslinked quattroGels containing gelatin and fibrin in a 20∶1 ratio.

Because the specific combination of all four additives in quattroGels - not the individual components themselves – revealed new biological features, it could not be excluded that thrombin generated repulsive factors from the gelatin. Such a phenomenon is evident for the endothelial membrane protein TEM5/GPR124, which is an adhesion G-protein-coupled receptor containing a cryptic (inactive) RGD motif in its extracellular domain. Thrombin cleavage and subsequent shedding of TEM5/GPR124 provides a biologically active RGD peptide which may function as soluble a- or antagonist of integrins, and thus modulates angiogenesis [37].

In addition to biological and chemophysical parameters, Forget et al. (2013) emphasized the critical role in how inductive biological signals are presented to endothelial cells. By inducing a switch in the secondary structure from α-helix to β-sheet through carboxylation, agarose gels with attached RGD peptides can selectively instruct endothelial cells to polarize and form vessel-like structures in vitro. This cell differentiation has been attributed to a *mechanobiology* mechanism based on the spatial advantageous presentation of secondary additives such as RGD on β-sheet agarose [38]. Conversely, the cell-specific effects of quattroGel originated potentially from the crosslinking of fibrin and gelatin which could disarrange cell-binding domains of both matrix proteins and consequently, counteract matrix-cell signaling essential for differentiation of neurons and endothelial cells.

Neurite extension of DRGs in homogeneous fibrin gels appears to be restricted and dependent on fibrinolysis by serine proteases [34]. However, due to the low content of fibrin (0.3%) in quattroGels, it remains questionable, whether a simple matrix molecule effect is responsible for the observed cell-type specificity. Similarly, it is unlikely that the proteolytical activity of thrombin accounted for axon inhibition. Although thrombin causes neurite retraction in neuroblastoma cells through activation and cleavage of cell surface thrombin receptors and subsequent protein kinase stimulation [39], in previous experiments we had no indication that the applied thrombin concentration in fibrin gels had neurite repulsive effects.

### Chondrogenesis

Our observation that chondrocytes accept hydrogel containing gelatin or fibrin as instructive microenvironment is in agreement with reports on macroporous poly(hydroxyethyl methacrylate)-gelatin cryogels [40] and e.g. fibrin-alginate constructs [41]. In contrast to monolayer cultures, chondrocytes with their glycolyse-based energy metabolism do not lose their spheroid phenotype in 3D hydrogels, where oxygen tension can be as low as 1% and still favor chondrocytic differentiation. Indeed, in quattroGels cell differentiation was initiated towards a chondrocytic phenotype.

## Conclusion

In spite of gelatin and fibrin being probably among the most intensively studied biomaterials, no attempt has been made to investigate their biological impact as blend material. Therefore, our data are most likely the first to reveal that a distinct component combination provides unexpected properties favoring a sessile cell type (chondrocytes) with simultaneous repulsion of motile cells (endothelial cells and neurons/axons). Receptor expression profiles could possibly provide some clue about the underlying mechanisms. *Mechanobiology* focusing on spatial presentation of matrix protein domains after fibrin-gelatin crosslinking might add to the specificity. In addition, the proteolytic and transamidase activities of transglutaminase and thrombin in the applied concentrations could generate new matrix entities or biogenic fragments. Besides the biological reasoning, material characteristics were likely to affect cell responses. Rheometric measurements demonstrated with 0.86 kPa a somewhat smaller storage modulus of quattroGel in comparison to gelatin gel. Nevertheless, we would have expected also quattroGel to be suitable for neurite outgrowth because on different fibronectin hydrogels neurite extension is evident within a broad range of G′ values 0.1 - 10 kPa. Another notable aspect is that the softer quattroGel proved to be much more resistant to resorption after subcutaneous implantation, though the opposite could have been predicted. In summary, the discussion highlights the complexity of the phenomena and possibly the likelihood of combinatorial effects. Although the causal links still need to be unraveled in the future, the observed cell specificity promises to open a novel avenue in implant designs for regenerative medicine.
